# Phillyrin for COVID-19 and Influenza Co-infection: A Potential Therapeutic Strategy Targeting Host Based on Bioinformatics Analysis

**DOI:** 10.3389/fphar.2021.754241

**Published:** 2021-11-04

**Authors:** Yanni Lai, Tiantian Han, Zizhao Lao, Geng Li, Jianyong Xiao, Xiaohong Liu

**Affiliations:** ^1^ School of Basic Medical Sciences, Guangzhou University of Chinese Medicine, Guangzhou, China; ^2^ Shenzhen Hospital of Integrated Traditional Chinese and Western Medicine, Shenzhen, China; ^3^ Laboratory Animal Center, Guangzhou University of Chinese Medicine, Guangzhou, China; ^4^ Research Center of Integrative Medicine, School of Basic Medical Sciences, Guangzhou University of Chinese Medicine, Guangzhou, China; ^5^ The First Affiliated Hospital of Guangzhou University of Chinese Medicine, Guangzhou, China

**Keywords:** phillyrin, Covid-19, influenza, co-infection, bioinformatics analysis

## Abstract

**Background:** The risk of co-epidemic between COVID-19 and influenza is very high, so it is urgent to find a treatment strategy for the co-infection. Previous studies have shown that phillyrin can not only inhibit the replication of the two viruses, but also has a good anti-inflammatory effect, which is expected to become a candidate compound against COVID-19 and influenza.

**Objective:** To explore the possibility of phillyrin as a candidate compound for the treatment of COVID-19 and influenza co-infection and to speculate its potential regulatory mechanism.

**Methods:** We used a series of bioinformatics network pharmacology methods to understand and characterize the pharmacological targets, biological functions, and therapeutic mechanisms of phillyrin in COVID-19 and influenza co-infection and discover its therapeutic potential.

**Results:** We revealed potential targets, biological processes, Kyoto Encyclopedia of Genes and Genomes (KEGG) pathways, and upstream pathway activity of phillyrin against COVID-19 and influenza co-infection. We constructed protein–protein interaction (PPI) network and identified 50 hub genes, such as MMP9, IL-2, VEGFA, AKT, and HIF-1A. Furthermore, our findings indicated that the treatment of phillyrin for COVID-19 and influenza co-infection was associated with immune balance and regulation of hypoxia-cytokine storm, including HIF-1 signaling pathway, PI3K-Akt signaling pathway, Ras signaling pathway, and T cell receptor signaling pathway.

**Conclusion:** For the first time, we uncovered the potential targets and biological pathways of phillyrin for COVID-19 and influenza co-infection. These findings should solve the urgent problem of co-infection of COVID-19 and influenza that the world will face in the future, but clinical drug trials are needed for verification in the future.

## Introduction

The Coronavirus disease 2019 (COVID-19), caused by SARS-CoV-2, rapidly spread and by March 11, 2020, the World Health Organization (WHO) declared COVID-19 a global pandemic. COVID-19 which has caused over 3 million deaths and has infected 140 million people since December 2019, poses a threat to the sustainability of public health systems worldwide, and has become the most ruinous outbreak since the H1N1 influenza in 1918 (Javelle and Raoult, 2021). Like influenza, COVID-19 is a febrile illness, sharing the same routes and means of transmission as influenza, which with clinical manifestations of an influenza-like illness (ILI) yet test negative for influenza (Lai, et al., 2020).

Influenza is an infectious disease of the respiratory tract caused by the influenza virus. In the 20th century, there have been at least four influenza pandemics: the Spanish pandemic in 1918, the H2N2 Asian pandemic in 1957, the H3N2 Hong Kong pandemic in 1968, and the H1N1 pandemic in 2009 (Morens and Taubenberger, 2018). The worst flu pandemic was the Spanish pandemic of 1918, which caused 5.1 billion deaths in 8 months (Morens and Taubenberger, 2018). Seasonal influenza epidemics and occasional pandemics are still public health concerns around the world.

In the foreseeable future, the co-circulate of COVID-19 and influenza is a problem that scientists cannot ignore. In fact, many countries have adopted comprehensive and long-term public health measures to prevent and manage the co-transmission of the two (Solomon et al., 2020; Soo et al., 2020). Studies have shown that co-infection of COVID-19 and influenza is fatal, and both are characterized by pneumonia and severe acute respiratory failure (Sockrider et al., 2020). In addition to the damage caused by the pathogenic factors themselves, the cytokine storm caused by the imbalance of the body’s immune function is also one of the important factors leading to the death of patients. In fact, the cytokine storm induced by influenza and COVID-19 has great similarities in pathological processes and other aspects: higher levels of cytokine and chemokine production have been found in both diseases, and the key mediators of cytokine storms are also highly similar, including IL-6, IL-1β, TNF-α, IL-10, and IP-10 (Jamilloux et al., 2020; Guo and Thomas, 2017); coagulation dysfunction and diffuse intravascular coagulation are closely related to the cytokine storm caused by the two viral infections (Yang and Tang, 2016; Mangalmurti and Hunter, 2020); in addition, SARS-CoV-2 and influenza virus infection can induce the activation of NLRP3 inflammasome (Chen et al., 2019). Studies have shown that virus-derived cytokine storm syndrome seems to have a common pathogenesis of immune response imbalance, increased inflammation, and T cell reduction and functional failure (Sun et al., 2020).

Although the COVID-19 vaccine has begun worldwide, it will take a long time to safely achieve herd immunity through vaccination. Therefore, the development of small-molecule drugs for the prevention or treatment of COVID-19 is still an urgent matter. A recent study shows that Molnupiravir, an oral broad-spectrum antiviral agent that is currently in phase II/III clinical trials, exerts marked inhibitory activity to SARS-CoV-2 *in vivo*, and the results of this study are undoubtedly exciting (Malone and Campbell, 2021; Wahl et al., 2021). But for the challenge of co-infection of influenza and SARS-CoV-2, we still need to further discover and develop drugs, especially the discovery of drugs that have better therapeutic effects on COVID-19 and influenza.

Therefore, we try to find potential compounds that can simultaneously regulate the excessive immune response caused by SARS-COV-2 and/or IAV from the perspective of the body’s immune balance. Surprisingly, we noticed that phillyrin has not only been shown to inhibit the replication of SARS-COV-2 or influenza virus, but also has anti-inflammatory effects (Wang et al., 2010; Qu et al., 2016; Chen et al., 2017; Wang et al., 2018; Ma et al., 2020; Zhang et al., 2021). Phillyrin exerts effective anti-SARS-COV-2 virus activity *in vivo* E6 cells (IC_50_ = 63.90 μg/ml) (Ma et al., 2020). Phillyrin is the main active ingredient of the traditional Chinese medicine Forsythia Suspensa. Forsythia Suspensa has the effects of clearing away heat, detoxifying, and reducing swelling and congestion. In China, it is often used to treat the “common cold” (Qu et al., 2016). Hence, in this study, we use bioinformatics methods to explain the key genes and biological pathways of phillyrin against co-infection of COVID-19 and influenza from the perspective of the body’s immune response triggered by pathogenic factors. According to preliminary analysis results, phillyrin is expected to be a candidate drug for the treatment of co-infection with COVID-19 and influenza.

## Materials and Methods

### Identification of COVID-19/Influenza Related Targets

COVID-19/influenza related targets were searched from several databases: GeneCards (https://www.genecards.org), CTD (http://ctdbase.org), and COVID-19 DisGeNET data collection (https://www.disgenet.org/covid/diseases/summary/). Species all selected *homo sapiens*.

In addition, we also collected COVID-19/influenza related targets from GEO database (https://www.ncbi.nlm.nih.gov/geo) by analyzing the GSE dataset. The transcriptomic RNA-seq data for COVID-19 including GSE147507 and GSE164805, and GSE147507 and GSE101702 for influenza. The criteria of differentially expressed genes (DEGs) were as follows: FDR < 0.05, and |log2FC| ≥ 2.

### Identification of Potential Targets for Phillyrin

The potential targets of phillyrin were predicted from Pharm Mapper (http://www.lilab-ecust.cn/pharmmapper/), ETCM (http://www.tcmip.cn/ETCM/index.php/Home/), SwissTargetPrediction (http://www.swisstargetprediction.ch/), and BindingDB (http://www.bindingdb.org/bind/index.jsp) databases. In Pharm Mapper database, “Human Protein Targets Only” was selected and the maximum generated conformations was set as 300. The parameter settings of ETCM, SwissTargetPrediction, and BindingDB databases were all selected by default.

Then, the overlap targets between phillyrin and COVID-19/influenza were obtained by jvenn online tools (http://jvenn.toulouse.inra.fr/app/example.html).

### Protein-Protein Interaction Network Analysis

Protein–protein interaction (PPI) analysis was carried out by STRING (https://www.string-db.org). The PPI network of the top 50 genes by degree ranking was constructed and visualized by Cytoscape 3.8.2 software (https://cytoscape.org) cytoHubba tool.

### Pathway and Gene Ontology Enrichment Analysis

To understand the related biological processes, pathway enrichment and gene ontology term enrichment analysis was conducted with the database for Annotation, Visualization and Integrated Discovery (DAVID, version 6.8) (https://david.abcc.ncifcrf.gov). Gene ontology clusters enrichment was carried out by Coronascape (http://coronascape.org) (Zhou et al., 2019). The biological processes with *p*-value < 0.05 were selected. Species all selected *homo sapiens*.

### Inference of Upstream Pathway Activity

To infer the mechanism origin of abnormal transcriptome regulation, we predict the upstream activation or inhibition of the overlap targets between phillyrin and COVID-19/influenza with the SPEED2 online tool (https://speed2.sys-bio.net). The “bates test” was selected as test statistics for enrichment.

## Results

### Potential Targets Identification of COVID-19, Influenza, and Phillyrin

**FIGURE 1:**
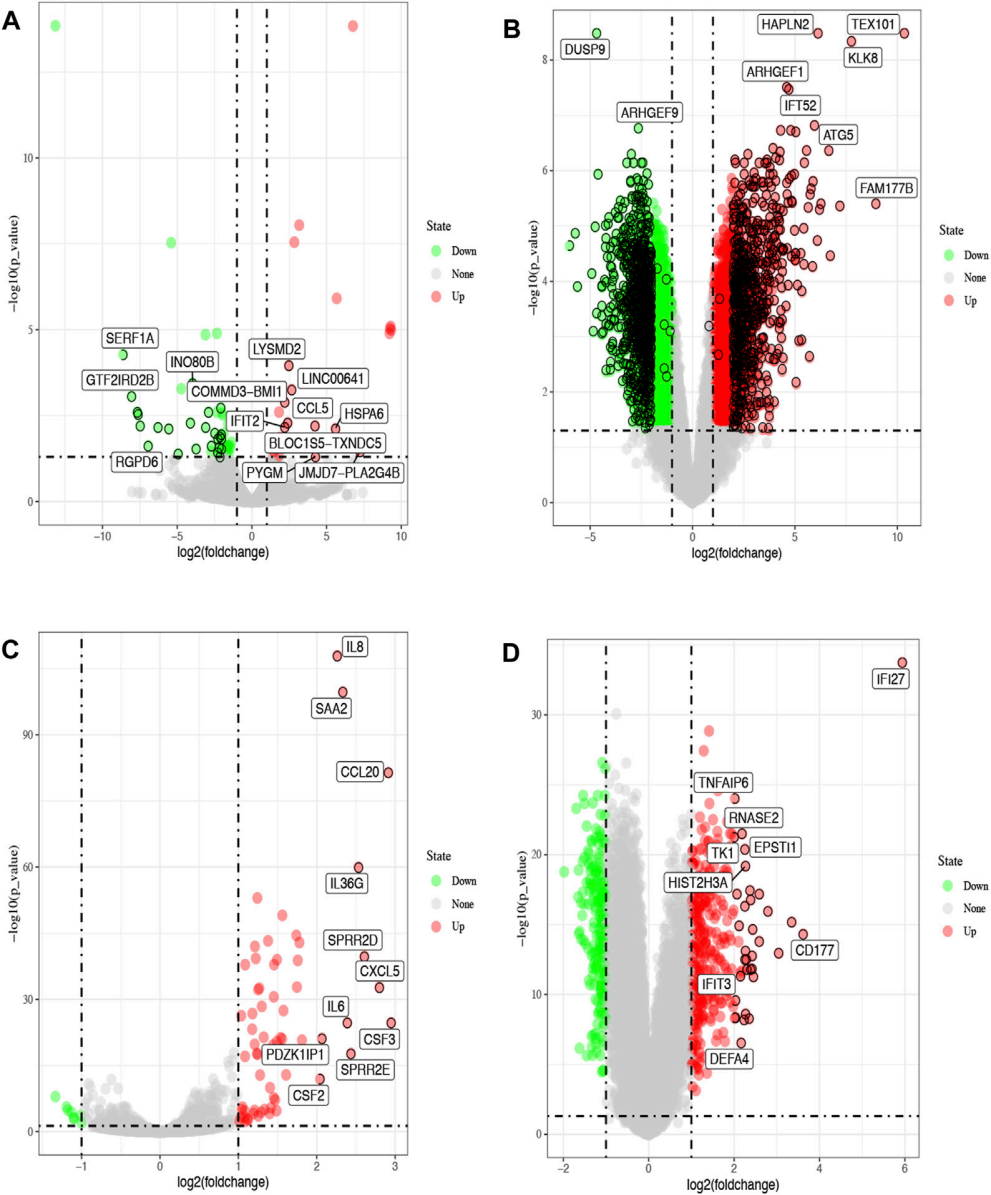
Volcano plots of differentially expressed genes (DGEs) from the GEO database. The red dots represent up-regulated genes, green represent down-regulated (FDR<0.05, |log2FC|>2). Differentially expressed genes of COVID-19 from GSE147507 **(A)** and GSE164805 **(B)**. Differentially expressed genes of influenza from GSE147507 **(C)** and GSE101702 **(D)**.

We first obtained 1688 and 69 DEGs for COVID-19 and influenza through analyzing GSE dataset, respectively. GSE147507 contained 3 samples of human bronchial epithelial cells (NHBE) infected SARS-CoV-2, and 3 samples of human lung adenocarcinoma cells (A549) infected SARS-CoV-2. GSE164805 contained the whole genome transcriptome to peripheral blood mononuclear cells (PBMCs) taken from 5 severe and 5 mild COVID-19 patients as well as 5 healthy controls. GSE147507 contained 3 samples of human bronchial epithelial cells (NHBE) infected with influenza A, and 3 samples of human lung adenocarcinoma cells (A549) infected with influenza A. GSE101702 contained peripheral blood samples taken from 63 moderate and 44 severe IAV patients as well as 52 healthy controls. The distribution of DEGs was illustrated by volcano plots as shown in Figures 1A–D. The clinical attributes including demography and blood routine examination of GSE164805 and GSE101702 are provided in Supplementary Table S1.

On the other side: we collected 8117 genes related to COVID-19 from GeneCards, CTD, and COVID-19 DisGeNET data collection databases, and 10,188 genes related to influenza from GeneCards and CTD databases. Finally, after removal of the duplicate genes, a total of 9186 related COVID-19 genes and 10,215 related influenza genes were collected.

**FIGURE 2:**
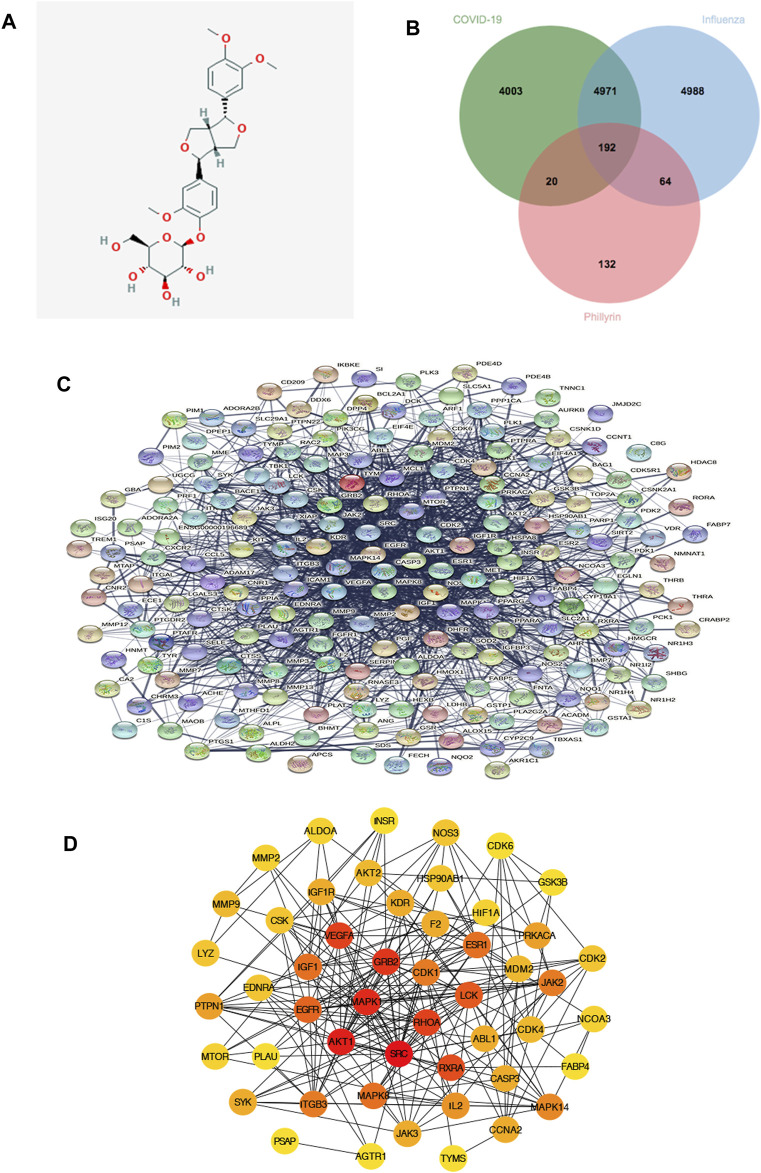
Protein-protein interaction (PPI) network of phillyrin against influenza and COVID-19. **(A)** The structure of phillyrin. **(B)** The intersection of phillyrin targets and disease targets. **(C)** 192 relevant overlapping genes. **(D)** The top 50 genes by degree ranking in the network.

By employing four available resources, namely, the Pharm Mapper, ETCM, SwissTargetPrediction, and BindingDB databases, we obtained 319, 4, 100, and 66 targets related to phillyrin, respectively, after removing duplication, and we finally collected 408 unique targets. The 2D structure of phillyrin was shown in Figure 2A. Then, we used jvenn online tools to obtained 192 overlapping targets between phillyrin and COVID-19/influenza (Figure 2B) (Supplementary Table S2).

### Protein-Protein Interaction Interation and Network Analysis

The 192 overlapping targets were imported into STRING database to build the PPI network and the PPI network is shown in Figure 2C. Then the top 50 genes by degree ranking in the PPI network were obtained and visualized by the cytoHubba tool (Figure 2D), including MMP9, MMP2, ALDOA, INSR, NOS3, CDK6, GSK3B, CDK2, NCOA3, FABP4, MAPK14, CCNA2, TYMS, AGTR1, PSAP, SYK, MTOR, PTPN1, LYZ, EDNRA, CSK, IGF1R, AKT2, HSP90AB1, HIF1A, PRKACA, JAK2, CDK4, CASP3, IL2, JAK3, ITGB3, PLAU, EGFR, IGF1, VEGFA, KDR, F2, ESR1, MDM2, ABL1, RXRA, MAPK8, AKT1, MAPK1, GRB2, CDK1, LCK, RHOA, and SRC, which indicates that these targets might be the core targets playing a pivotal role in the network.

### Pathway and Gene Ontology Enrichment Analysis of Overlapping Targets

**FIGURE 3:**
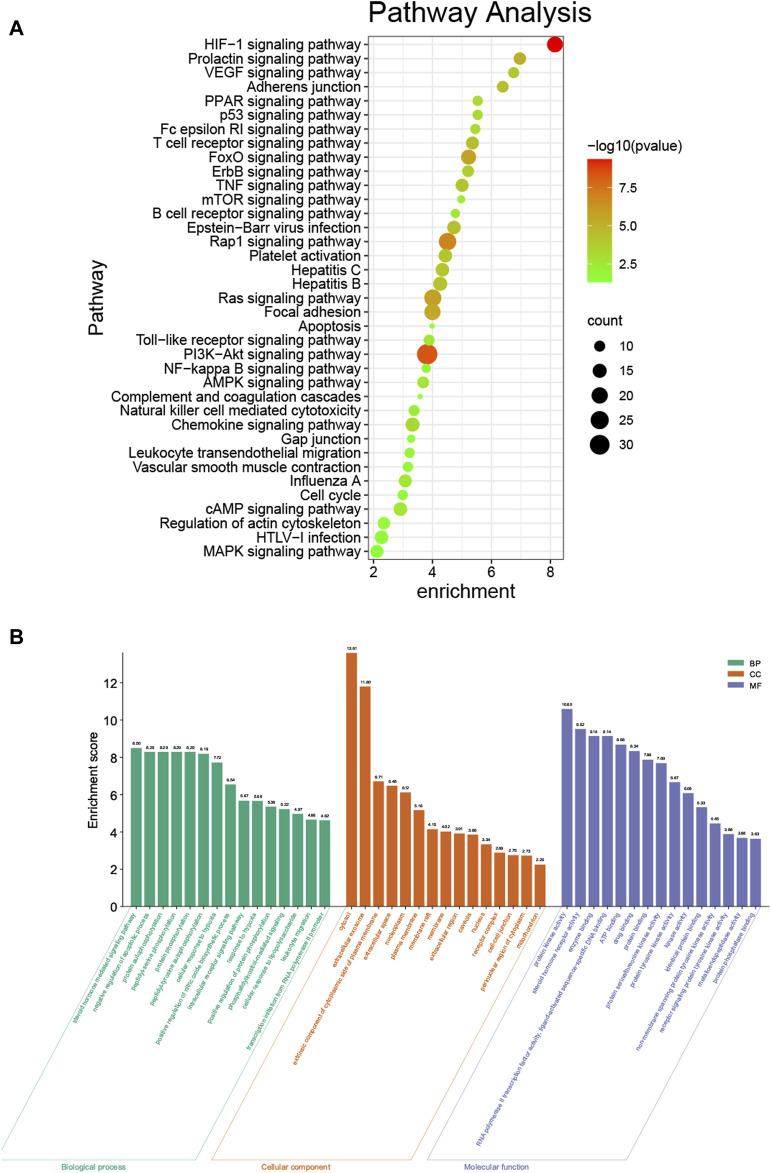
KEGG and GO enrichment analysis. **(A)** KEGG enrichment analysis bubble chart by DAVID. **(B)** GO enrichment analysis by DAVID.

To explore the biological functions of phillyrin in COVID-19/influenza, KEGG enrichment and GO enrichment analysis were performed on the 192 candidate targets by DAVID. The pathway enrichment bubble chart is shown in Figure 3A, which contained 37 terms. The top 15 pathways ranked by FDR were: HIF-1 signaling pathway, PI3K-Akt signaling pathway, Rap1 signaling pathway, thyroid hormone signaling pathway, estrogen signaling pathway, Ras signaling pathway, FoxO signaling pathway, focal adhesion, prolactin signaling pathway, T cell receptor signaling pathway, Epstein-Barr virus infection, Adheren junction, TNF signaling pathway, Hepatitis B, and VEGF signaling pathway (Supplementary Table S3).

The top 15 (with the FDR) enriched GO terms of the biological process category are shown in Figure 3B. The most abundant GO terms were steroid hormone mediated signaling pathway (GO:0043401), cytosol (GO:0005829), and protein kinase activity (GO:0004672), for biological process (BP), CC (cellular component), and MF (molecular function), respectively (Supplementary Table S4).

### Gene Ontology Clusters Enrichment Analysis of Overlapping Targets

**FIGURE 4:**
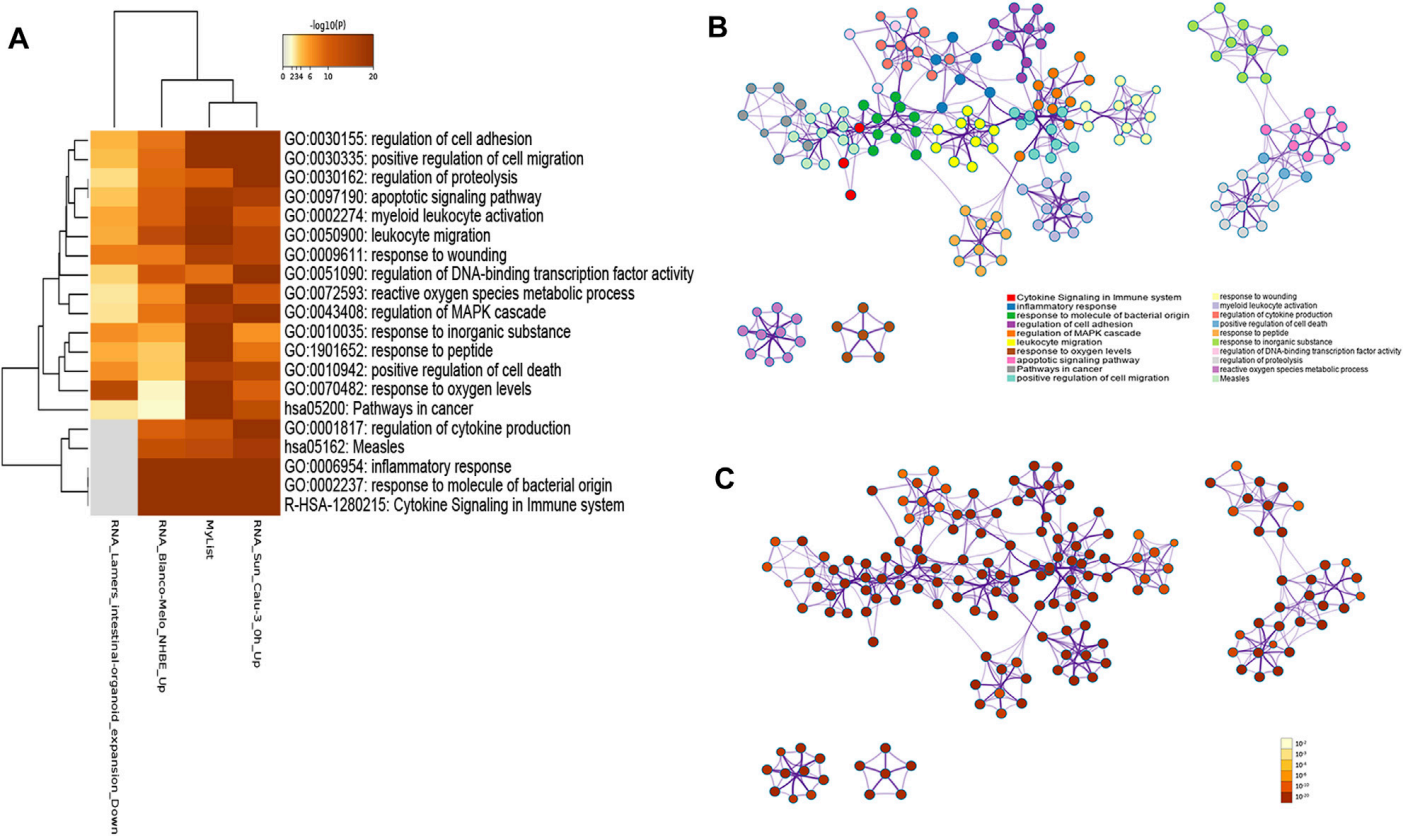
**(A)** Heatmap of the top 20 enriched GO terms for 192 genes by Metascape, colored by *p*-values. The darker the color, the higher the ranking. **(B)** Network of enriched terms colored by cluster ID, where nodes that share the same cluster ID are typically close to each other. **(C)** Network of enriched terms colored by *p*-value, where terms containing more genes tend to have a more significant *p*-value.

To investigate gene functions in each gene cluster, we used Metascape to carry out GO enrichment and GO clusters analysis with the following ontology sources: KEGG Pathway, GO Biological Processes, Reactome Gene Sets, Canonical Pathways, CORUM, TRRUST, DisGeNET, PaGenBase, and COVID. Heatmap of the top 20 (with the *p*-values) enriched GO terms of the biological process category is shown in Figure 4A, including cytokine signaling in the immune system, inflammatory response, response to molecules of bacterial origin, regulation of cell adhesion, regulation of MAPK cascade, and so on (Supplementary Table S5). To further capture the relationships between the terms, we used Metascape to perform GO clusters analysis. A subset of enriched terms has been selected and rendered as a network plot, where terms with a similarity >0.3 are connected by edges. The network was visualized using Cytoscape 3.8.2, where each node represents an enriched term and was colored first by its cluster ID (Figure 4B) and then by its *p*-value (Figure 4C).

### Quality Control and Association Analysis of Overlapping Targets

**FIGURE 5:**
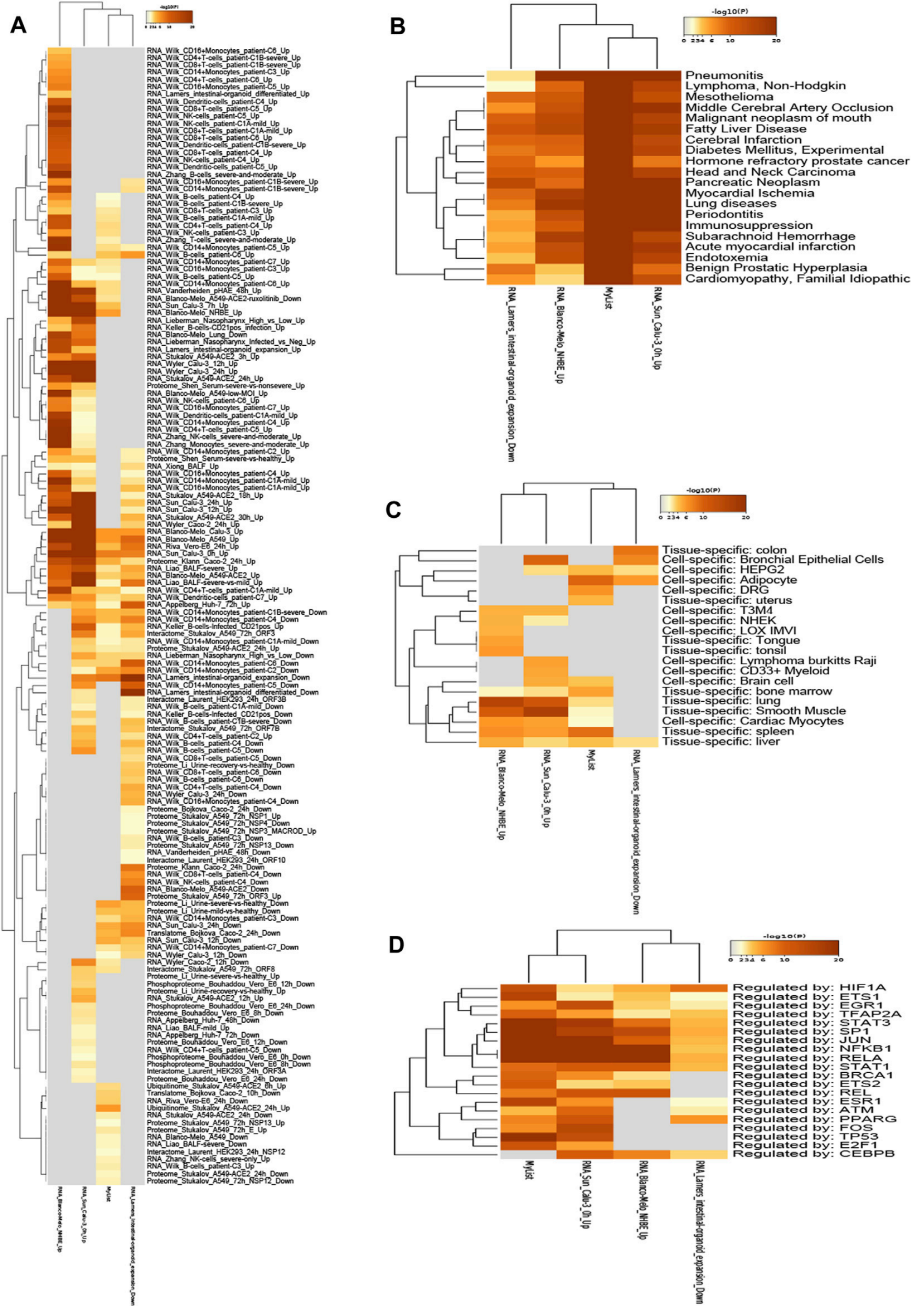
Heatmap of 192 genes enrichments are identified in the following ontology categories: COVID, DisGeNET, PaGenBase, TRRUST, colored by *p*-values. The darker the color, the higher the ranking. **(A)** Enrichment analysis result in COVID. **(B)** Enrichment analysis result in DisGeNet. **(C)** Enrichment analysis result in PaGenBase. **(D)** Enrichment analysis result in TRRUST.

To further study the association between 192 overlapping targets, genes enrichments were identified in the following ontology categories: COVID, DisGeNET, PaGenBase, and TRRUST (Figures 5A–D) (Supplementary Table S6). Terms were collected and grouped into clusters based on their membership similarities (*p*-value < 0.01, a minimum count of 3, and an enrichment factor >1.5). As shown in Figure 5A, there were 157 terms identified in COVID database, including RNA_Wilk_CD16 + Monocytes_patient-C6_Up, RNA_Wilk_CD4+T-cells_patient-C1B-severe_Up, RNA_Wilk_CD8+T-cells_patient-C1B-severe_Up, RNA_Wilk_CD14 + Monocytes_patient-C3_Up, RNA_Wilk_CD4+T-cells_patient-C6_Up, and so on. The top 20 terms identified in DisGeNET database are shown in Figure 5B, and the top 5 terms are as follows: pneumonitis, lymphoma (non-Hodgkin), mesothelioma, middle cerebral artery occlusion, and malignant neoplasm of the mouth. GO analysis heatmap of 192 targets in PaGenBase is shown in Figure 5C, containing colon (tissue-specific), bronchial epithelial cells (cell-specific), HEPG2 (cell-specific), adipocyte (cell-specific), DRG (cell-specific), and so on. Transcription factors related to 192 targets were analyzed in the TRRUST database (Figure 5D), the top 5 terms, namely, HIF1A, ETS1, EGR1, TFAP2A, and STAT3.

### Upstream Pathway Analysis of Overlapping Targets

**FIGURE 6:**
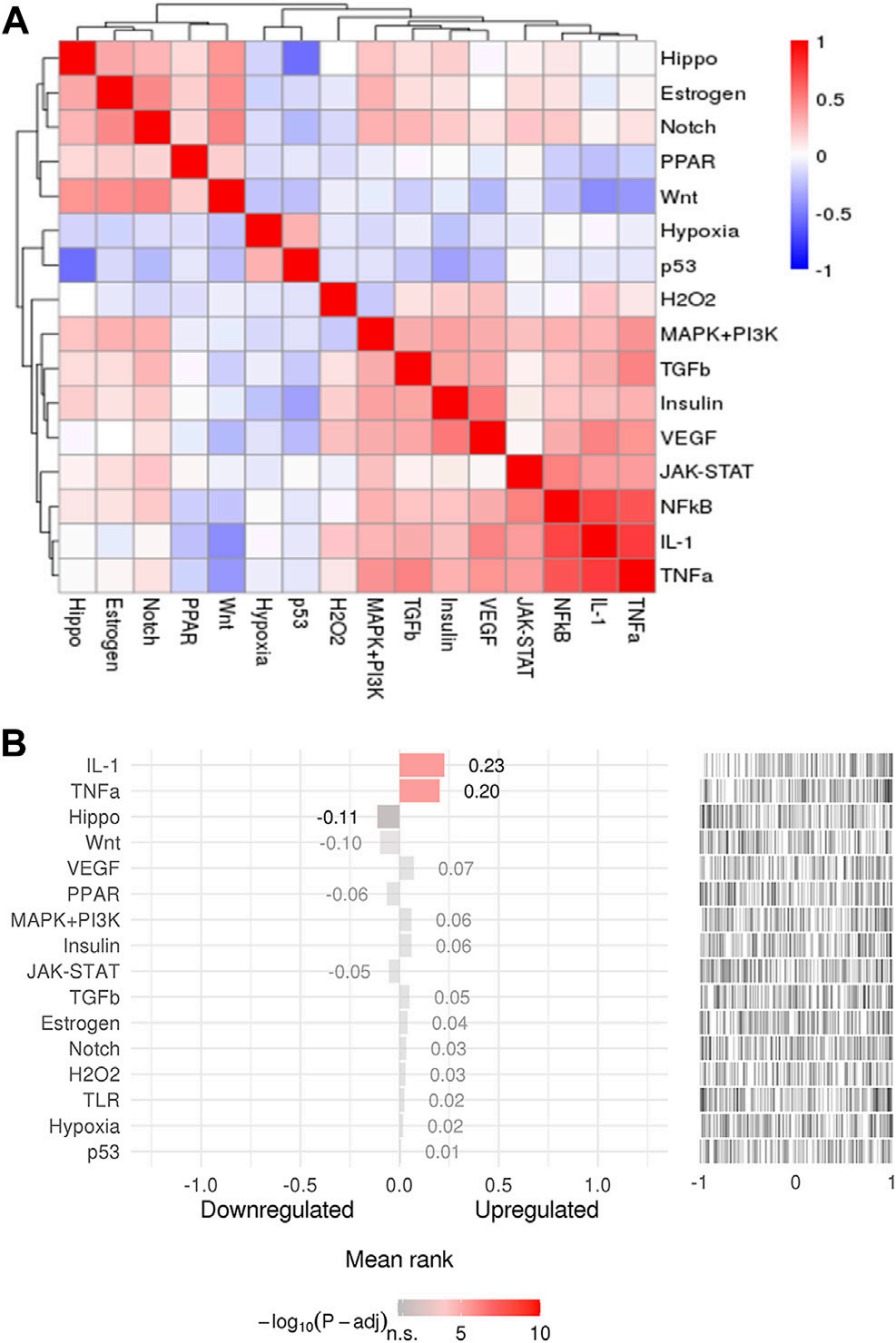
**(A)** The pathway clusters for 192 genes. **(B)** Pathway activity ranking (adjusted *p*-value < 0.05). The colors indicated adjusted *p*-value, and the brighter the color, the higher the ranking.

To explore the upstream pathway of the overlapping targets, the SPEED2 tool was used to perform. As shown in Figures 6A,B, there were 12 upstream pathways up-regulated related to the overlapping targets, while only 4 upstream pathways down-regulated (Supplementary Table S7). TNFα, IL-1, VEGF, MAPK + PI3K, insulin, TGFb, estrogen, notch, H_2_O_2_, TLR, hypoxia, and P53 pathways were inferred up-regulated with overlapping targets, and Hippo, Wnt, PPAR, and JAK-STAT pathways were inferred down-regulated in upstream (Figure 6B). Colors indicated the adjusted *p*-value, and the ranked lists about activity were determined by the absolute *p*-value, the brighter the color, the higher the ranking.

## Discussion

The annual influenza epidemic has a significant impact on the health care system worldwide. With the COVID-19 pandemic in 2019, clinicians are faced with a second respiratory virus whose morbidity and mortality are several-fold higher than that of influenza. Therefore, the imminent threat of concurrent influenza and COVID-19 pandemics is a major concern for public health officials and clinicians.

Although there are antiviral drugs and influenza vaccines for influenza viruses in clinical practice, the mutation of the virus and the emergence of drug resistance have made the original treatments limited. At present, there is no anti-SARS-COV-2 drug that can be used, and the population coverage of vaccines for herd immunity will still take some time. Therefore, for the host immune target, the development of drugs that can simultaneously treat COVID-19 and influenza co-infection is a potential treatment strategy. In this study, we explored whether phillyrin has a potential protective effect against SARS-COV-2 and influenza virus co-infection; to clarify this issue, we predicted the potential targets and biological pathways for co-infection with SARS-COV-2 and influenza virus through integrated bioinformatics. Preliminary studies have shown that phillyrin is expected to be a candidate drug for the treatment of SARS-COV-2 and influenza virus co-infection.

Our research showed that phillyrin might target 192 common targets of COVID-19 and influenza, which means that these targets were potential targets for phillyrin to function. Targets in the PPI network were extracted and analyzed, and the results suggested that 50 genes, including MMP9, MMP2, ALDOA, INSR, NOS3, CDK6, GSK3B, CDK2, NCOA3, FABP4, MAPK14, CCNA2, TYMS, AGTR1, PSAP, SYK, MTOR, PTPN1, LYZ, EDNRA, CSK, IGF1R, AKT2, HSP90AB1, HIF1A, PRKACA, JAK2, CDK4, CASP3, IL2, JAK3, ITGB3, PLAU, EGFR, IGF1, VEGFA, KDR, F2, ESR1, MDM2, ABL1, RXRA, MAPK8, AKT1, MAPK1, GRB2, CDK1, LCK, RHOA, and SRCA, were the core genes in the network. Previous research indicated that the core genes may be crucial to the treatment mechanism. For example, the increase in IL-2, MMP9, and VEGFA is related to the mortality of COVID-19 patients (Young et al., 2020; Abers et al., 2021). It has been reported that circulating MMP-9 increases significantly and early in patients with COVID-19 respiratory failure (Ueland et al., 2020). In acute lung injury, MMP-9 released from neutrophils will promote the inflammation and degradation of the alveolar capillary barrier, and further stimulate the migration and final structure of the alveoli (Davey et al., 2011). In influenza, the virulent influenza virus infection is characterized by a large number of cell infiltration and severe lung pathology, which is related to the production of oxidative stress and MMP-9 (Rojas-Quintero et al., 2018). MMP-9 can cleave various proteins to regulate inflammation and injury responses (Bradley et al., 2012). MMP-9 can mediate the migration of neutrophils into the airway in response to Toll-like receptor signals induced by influenza viruses (Callahan et al., 2021). The main characteristics of SARS-CoV-2 induced pulmonary complications include the overexpression of pro-inflammatory chemokines and cytokines, which can lead to a “cytokine storm.” After SARS-CoV-2 infects Calu-3 human lung epithelial cells, the pro-inflammatory chemokines CXCL9, CXCL10, and CXCL11 are up-regulated in an AKT-dependent manner (Li et al., 2021). Furthermore, complete transcriptome RNA sequencing of the peripheral blood of COVID-19 patients revealed that AKT1 is one of the central genes in the differential gene PPI network (Casalino-Matsuda et al., 2020). In influenza, hypercapnia (HC) is a risk factor for mortality in patients with severe acute and chronic lung diseases, suppressing macrophage antiviral activity and increasing mortality of influenza A infection via AKT1 (Taniguchi-Ponciano et al., 2021). The highly contagious SARS-CoV-2 virus mainly attacks lung tissue and impairs gas exchange, leading to acute respiratory distress syndrome (ARDS) and systemic hypoxia. Hypoxia and cytokine storm are the main pathophysiological characteristics of COVID-19, and the prelude to multiple organ failure and mortality. Hypoxia-inducible factor 1α (HIF-1α) is involved in the activation of pro-inflammatory cytokine expression and the subsequent inflammatory process, which makes it a potential molecular marker of the severity of COVID-19 (Jahani et al., 2020; McElvaney et al., 2020; Serebrovska et al., 2020). In addition, recent studies have shown that hypoxia-inducible factor 1-alpha (HIF-1α) is associated with the production of proinflammatory molecules in the severe pneumonia caused by H1N1 infection (Guo et al., 2017).

The results of the KEGG enrichment of 192 targets indicated that phillyrin might mainly regulate HIF-1 signaling pathway, PI3K-Akt signaling pathway, and Ras signaling pathway to reduce the inflammation in the lungs caused by SARS-CoV-2 and influenza virus co-infection. As well as we know, hypoxia and cytokine storm are highly correlated influencing factors with the severity of COVID-19 and severe influenza. Hypoxia is a common feature of inflammation, and HIF-1α transcription factor promotes inflammation by up-regulating genes containing HRE in pro-inflammatory immune cells (including neutrophils, DCs, and macrophages) (McElvaney et al., 2020). Its expression and activity are strictly regulated by oxygen content, and it is considered an “inflammation switch.” Inappropriate reactions can lead to tissue destruction, blood vessel damage, and organ failure, although proper inflammation helps eradicate infectious pathogens and maintain tissue integrity (Marchetti, 2020). The HIF-1 signaling pathway is expected to become one of the targets for effective treatment of influenza and COVID-19 co-infection in the future.

PI3K-Akt signaling pathway plays an important role in the cell entry and immune response development of SARS-CoV-2 virus. After SARS-CoV-2 binds to ACE2, it activates CD147 and furin for endocytosis, which is regulated by PI3K-AKT signal transduction (Asselah et al., 2020). In addition, inhibiting the PI3K-AKT signaling pathway can inhibit the activation of activated protein-1 (AP-1) and nuclear factor kappa B (NF-KB), thereby reducing the expression of inflammatory cytokines (Yodkeeree et al., 2018).

RAS plays an important role in the occurrence and development of new respiratory infectious diseases such as SARS and influenza. RAS dysfunction is the key to ALI/ARDS in patients with virus infection (Gao et al., 2020). ACE2 is a key member of RAS, as the receptor of SARS-CoV-2, can assist the viruses to enter the body, reduce the level of ACE2, and cause RAS dysfunction (Hoffmann et al., 2020). After entering the body, H5N1, H7N9, and other viruses can inhibit the expression of ACE2 and increase the level of Ang II by binding to other receptors, thereby forcing RAS imbalance (Huang et al., 2014; Zou et al., 2014). They then activate the inflammatory pathway and innate immunity, a large number of cytokines, and chemokines act together to induce ALI and even ARDS. Therefore, some scholars believe that the use of renin angiotensin receptor antagonists may improve the inflammatory response, regulate immunity, maintain or restore the integrity of the pulmonary microvascular barrier, and ultimately reduce the mortality rate.

Phillyrin exhibited anti-virus (Wang et al., 2010; Ma et al., 2020), anti-inflammatory (Pan et al., 2014), antioxidant, and antibacterial (Qu et al., 2008) effects. A previous study has reported that phillyrin could inhibit novel coronavirus (SARS-CoV-2) and human coronavirus 229E (HCoV-229E) replication *in vitro*, and reduced the proinflammatory cytokines (TNF-α, IL-6, IL-1β, MCP-1, and IP-10) expression by regulating the activity of the NF-кB signaling pathway (Ma et al., 2020). In addition, phillyrin shows protective effects against influenza A *in vivo*, with significantly prolonging the mean survival time, reducing the lung index, decreasing the virus titers and interleukin-6 levels, reducing the expression of HA, and attenuating lung tissue damage (Qu et al., 2016). Moreover, phillyrin suppresses the expressions of IL-1β, IL-6, TNF-α, iNOS, and COX-2 in LPS-stimulated RAW264.7 macrophages *in vitro* by inhibiting JAK-STATs and p38 MAPKs signaling pathways and the production of ROS (Pan et al., 2014). We also have found that phillyrin reduces pulmonary inflammation via inhibiting MAPK and NF-κB pathways in an acute lung injury model induced by LPS (Zhong et al., 2013). Phillyrin not only has a direct antiviral effect, but also has multiple biological activities such as good anti-inflammatory and immune-regulating activities.

Our research shows that phillyrin can be used as one of the compound candidates for co-infection of SARS-CoV-2 and influenza virus, but there are some limitations. First, biological information analysis based on existing databases has the problems of inconsistent data processing standardization and the subjectivity of data set selection. Second, this research is based on the conclusions drawn from a series of texts and data analysis, and further *in vivo* and *in vitro* experiments are needed to support our research. Finally, this study mainly focuses on exploring the feasibility of phillyrin in the treatment of COVID-19 and influenza co-infection.

## Conclusion

In summary, the severity of pneumonia caused by SARS-COV-2 and influenza virus infection is closely related to the cytokine storm mediated by excessive inflammation. Controlling excessive inflammation and reducing the body’s immune damage is one of the important strategies for the treatment of COVID-19 and influenza. This study identified 192 common core targets and 25 biological pathways for phillyrin to treat co-infection of SARS-COV-2 and influenza virus through bioinformatics analysis. Combining the results of biosynthesis analysis and previous studies, we infer that phillyrin may mainly act on the HIF-1 signaling pathway, PI3K-AKT signaling pathway, and RAS signaling pathway to regulate the body’s immune and anti-inflammatory effects, thereby reducing the severity of diseases caused by SARS-COV-2 and influenza virus infections. These findings provide a theoretical and scientific basis for the further development of phillyrin to treat COVID-19 and influenza co-infection.

## Data Availability

The datasets presented in this study can be found in online repositories. The names of the repository/repositories and accession number(s) can be found in the article/Supplementary Material.
